# The Biological Efficacy of Natural Products against Acute and Chronic Inflammatory Diseases in the Oral Region

**DOI:** 10.3390/medicines5040122

**Published:** 2018-11-13

**Authors:** Toshiaki Ara, Sachie Nakatani, Kenji Kobata, Norio Sogawa, Chiharu Sogawa

**Affiliations:** 1 Department of Dental Pharmacology, Matsumoto Dental University, 1780 Gobara Hirooka, Shiojiri 399-0781, Japan; toshiaki.ara@mdu.ac.jp (T.A.); norio.sogawa@mdu.ac.jp (N.S.); 2 Faculty of Pharmacy and Pharmaceutical Sciences, Josai University, 1-1 Keyakidai, Sakado, Saitama 350-0295, Japan; s-nakata@josai.ac.jp (S.N); kobata@josai.ac.jp (K.K.); 3 Department of Dental Pharmacology, Okayama University Graduate School of Medicine, Dentistry and Pharmaceutical Sciences, 2-5-1 Shikata-cho, Okayama 700-8525, Japan

**Keywords:** inflammatory disease, stomatitis, periodontitis, anti-osteoclast activity, cepharanthin, herbal medicine, natural product, arachidonic acid cascade

## Abstract

The oral inflammatory diseases are divided into two types: acute and chronic inflammatory diseases. In this review, we summarize the biological efficacy of herbal medicine, natural products, and their active ingredients against acute and chronic inflammatory diseases in the oral region, especially stomatitis and periodontitis. We review the effects of herbal medicines and a biscoclaurin alkaloid preparation, cepharamthin, as a therapy against stomatitis, an acute inflammatory disease. We also summarize the effects of herbal medicines and natural products against periodontitis, a chronic inflammatory disease, and one of its clinical conditions, alveolar bone resorption. Recent studies show that several herbal medicines such as kakkonto and ninjinto reduce LPS-induced PGE${}_{2}$ production by human gingival fibroblasts. Among herbs constituting these herbal medicines, shokyo (*Zingiberis Rhizoma*) and kankyo (*Zingiberis Processum Rhizoma*) strongly reduce PGE${}_{2}$ production. Moreover, anti-osteoclast activity has been observed in some natural products with anti-inflammatory effects used against rheumatoid arthritis such as carotenoids, flavonoids, limonoids, and polyphenols. These herbal medicines and natural products could be useful for treating oral inflammatory diseases.

## 1. Introduction

Oral inflammatory disease is a general term for the inflammatory lesions developed in oral mucosa. The pathogenesis of oral inflammatory diseases is non-uniform due to the involvement of various factors—such as external and mechanical stimuli, the presence of microorganisms, and the overall physical conditions—that play a role in the onset of inflammation. There is a wide range of variations in the aspect of oral inflammatory diseases, and the aspect is unequal. Therefore, we construed the oral inflammatory diseases as a symptom of inflammation, and categorized them into acute and chronic inflammatory diseases. In the oral region, the representative example of acute inflammatory diseases is stomatitis (also named as oral mucositis), and that of chronic inflammatory diseases is periodontitis. Several Japanese herbal medicines (also known as kampo medicines) are clinically used for the treatment of inflammatory diseases. Recent reviews have summarized the therapeutic application of herbal medicines for oral diseases such as stomatitis and periodontitis [1]. For example, hangeshashinto (TJ-14) is used for inflammatory diseases such as acute or chronic gastrointestinal catarrh, nervous gastritis and stomatitis.

In this review, we aim to summarize the biological efficacy of herbal medicine, natural products, and their active ingredients against acute and chronic inflammatory diseases in the oral region, especially stomatitis and periodontitis.

## 2. Biological Efficacy of Natural Products against Acute Inflammatory Disease: Stomatitis

### 2.1. Stomatitis (Oral Mucositis)

Stomatitis is an inflammatory condition of the oral and oropharyngeal mucosa with both pain and ulcers in severe cases. The causes of stomatitis is classified into (1) bacterial or viral infection, (2) chemotherapy and/or radiation for the treatment of cancers, (3) autoimmune disease (such as lichen planus and pemphigus vulgaris), and (4) unknown (such as recurrent aphthous stomatitis). Recurrent aphthous stomatitis is a common condition characterized by the repeated formation of benign and non-contagious mouth ulcers (aphthae). However, the cause of aphthous stomatitis is still unknown.

### 2.2. Effect of Hangeshashinto on Stomatitis

Recently, clinical administration of herbal medicine, such as the treatment of recurrent aphthous stomatitis, has been increasing in Japan. Herbal medicines are chosen according to the patient’s condition, called “sho” (pattern), for example “excess pattern” or “deficiency pattern.” Among the herbal medicines, some products such as hangeshashinto (TJ-14), orengedokuto (TJ-15), orento (TJ-120), inchinkoto (TJ-135), byakkokaninjinto (TJ-34), juzentaihoto (TJ-48), and shosaikoto (TJ-9) are selected in the treatment against oral inflammatory diseases, including recurrent aphthous stomatitis, according to the patient’s pattern [2]. In addition, it seems that hangeshashinto is considered effective in the treatment of stomatitis caused by anti-tumor agents and radiation therapy [2].

In a preliminary study, rinsing with hangeshashinto reduced the grade of stomatitis [by Common Terminology Criteria for Adverse Events (CTCAE) version 4.0, National Cancer Institute, Bethesda, MD] [3]. Moreover, in a double-blind, placebo-controlled, random, phase II study, the rinsing of the oral cavity with hangeshashinto showed a trend to reduce the risk of chemotherapy-induced stomatitis in patients with gastric cancer [4]. In this study, hangeshashinto reduced the risk of grade 1 stomatitis but did not reduce those of more than grade 2 [4]. In a retrospective study, rinsing and gargling with hangeshashinto prevented grade 3/4 stomatitis induced by (chemo)radiation in patients with head and neck cancers (odds ratio = 0.21, 95% CI: 0.045–0.780, hangeshashinto: n=27, placebo: n=32) [5]. In addition, hangeshashinto also improved the rates of the treatment of stomatitis [5].

In an animal model, free intake of diet mixed with 2% hangeshashinto prevented radiation-induced mucositis within the buccal mucosa in hamsters [6]. In addition, hangeshashinto inhibited the infiltration of neutrophils and COX-2 expression in irradiated buccal mucosa [6]. Moreover, in an in vitro study using oral keratinocytes, hangeshashinto was suggested to be effective in the treatment of chemotherapy-induced stomatitis [7]. As just described, hangeshashinto is effective for the improvement of stomatitis although there is little evidence in in vivo and in vitro studies.

### 2.3. Effect of Cepharanthin${}^{®}$ on Stomatitis

A biscoclaurin alkaloid preparation, Cepharanthin${}^{®}$ (CE), has also been used for the cure of oral mucosal disease, such as recurrent aphthous stomatitis, leukoplakia, and oral lichen planus. CE is a drug product, prepared from extracts of *Stephania cephalantha* Hayata, and has been widely used for several decades to treat a range of acute and chronic diseases in Japan [8,9]. As CE is reported to elicit an anti-inflammatory effect and increase blood stem cell count, immuno-enhancing effects, and anti-allergic properties, it has seen clinical application against inflammatory diseases as well as post-radiation-therapy leukocytopenia, pit viper bite, alopecia areata, and bronchial asthma. Nakase et al. reported that the rate of excellent or moderate efficacy was 100% for aphthous stomatitis and 25.0% for reducing the size of oral lichen planus, and its efficacy for glossodynia was 83.4% by CE treatment with gargle-internal use (15 mg/day) for two weeks [10]. Moreover, Saki et al. also reported—regarding the efficacy of CE against these oral mucosal diseases— that the improvement rate by oral administration of CE (20 mg/day) for 4 weeks or more was 83.3% for aphthous stomatitis, 87.0% for oral lichen planus, 77.8% for glossodynia, and 80.0% for leukoplakia. In this case, they evaluated the clinical response and rated according to the assessment points such as the degrees of pain, ulcer, erosion, and erythema [11]. Taken together, it is considered that CE is beneficial in the cure of aphthous stomatitis, according to previous clinical reports [12].

**Figure 1:**
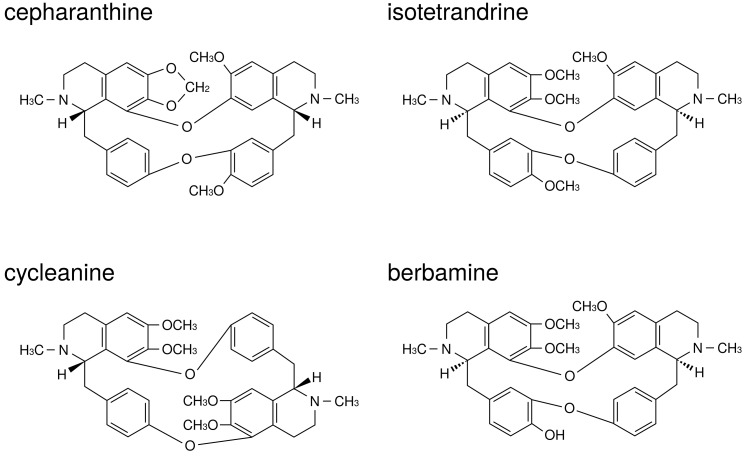
The structures of active ingredients in Cepharanthin${}^{®}$.

CE is a biscoclaurin alkaloid preparation, and the main active ingredients are four alkaloids: cepharanthine (26%), isotetrandrine (32%), berbamine (13%), and cycleanine (10%) (Figure 1). Using a mixture of these four active ingredients in CE exhibits almost an equal effect as that of CE [13,14]. Functional mechanisms of CE and its main active ingredients for inflammatory diseases have been reported in previous studies. For example, CE reduced the production of superoxide anion (O${}_{2}^{-}$) by neutrophils [15] and by macrophages [16], and decreased the levels of several types of reactive oxygen species (O${}_{2}^{-}$, H${}_{2}$O${}_{2}$, OH${}^{\cdot }$) by behaving as a reactive oxygen species (ROS) scavenger [17].

According to previous literatures corresponding to the application of the four main active ingredients, cepharanthine was reported to inhibit the synthesis of leukotriene B4 through the reduction of arachidonic acid release [18]. Moreover, each of the four main active ingredients reduced NO production by activated macrophages [19]. However, there was a difference in the efficacy against the O${}_{2}^{-}$ and TNF-α production among main active ingredients; the efficacy of cepharanthine and isotetrandrine seemed to be more than that of berbamine and cycleanine in the reduction of O${}_{2}^{-}$ production by neutrophils [20]. It was also reported that cepharanthine, isotetrandrine, and cycleanine, but not berbamine, significantly reduced the level of TNF-α or acute lethal toxicity induced by lipopolysaccaride (LPS) in mice [13,21]. Additionally, Matsuno et al. reported that the decreasing effect of O${}_{2}^{-}$ production through neutrophil stimulation by arachidonic acid and *N*-formylmethionine-leucyl-phenylalanine (FMLP) was more evident in cepharanthine than in opsonized zymogen [20]. This finding indicates the cell membrane to be a possible operating point of CE, and this hypothesis is supported in the following study by Sugiyama et al., who reported that cepharanthine could inhibit histamine release from mast cells through the stabilization of the membrane by decreasing membrane fluidity via interaction with the lipid bilayer of the cell membrane [22].

Interestingly, the pharmacological actions of CE on living bodies vary depending on the method of administration. We reported that the single injection of CE reduced the LPS-induced histidine decarboxylase (HDC) activity, although contrastingly, LPS-induced HDC activity in mice spleens increased after consecutive administration of CE [23]. Moreover, it was considered that mast cell was closely associated with this reduction of HDC activity, because LPS-induced HDC activity in mast-cell-deficient mice increased, but decreased in normal mice following a single administration of CE [23]. CE has immuno-enhancing effects as well as anti-inflammatory effects. The inhibition of mast cells may be closely related to the difference of CE action.

Conclusively, CE is considered to be an effective treatment of oral inflammatory diseases, such as recurrent aphthous stomatitis, through the reduction of various function in immunocytes closely related to inflammation.

## 3. Biological Efficacy of Natural Products against Chronic Inflammatory Disease; Periodontitis

### 3.1. Periodontitis

Periodontal disease (periodontitis) comprises a group of infections that leads to inflammation of the gingiva and destruction of periodontal tissues, and is accompanied by alveolar bone loss in severe clinical cases. The tissue destruction is the result of activation of the host’s immuno-inflammatory response to virulent factors such as LPS and peptidoglycan. In inflammatory responses and tissue degradation, prostaglandin E${}_{2}$ (PGE${}_{2}$), interleukin (IL)-6, and IL-8 play important roles. As PGE${}_{2}$ has several functions in vasodilation, the enhancement of vascular permeability and pain, and osteoclastogenesis induction, PGE${}_{2}$ participates in inflammatory responses and alveolar bone resorption in periodontitis [24].

Generally, periodontitis is a chronic inflammation, and the elimination of these virulent factors by initial preparation is very important for the treatment of periodontitis. However, during the acute advanced stage, non-steroidal anti-inflammatory drugs (NSAIDs) are administrated to improve gingival inflammation. In fact, many studies have demonstrated that systemic administration of acid NSAIDs prevented gingival inflammation and alveolar bone resorption in animals and humans [25]. However, acid NSAIDs are well known to have side effects such as gastrointestinal dysfunction and bronchoconstriction. Therefore, the usage of alternative agents is necessary for patients with gastrointestinal ulcer or bronchial asthma. Previously, we suggested that several herbal medicines are effective for the improvement of periodontitis. In this review, we focused on the anti-inflammatory effects of herbal medicines on mainly periodontitis —in particular, about the effects on human gingival fibroblasts (HGFs). In addition, we summarized the effects of ingredients in herbs and their mechanism against arachidonic acid cascade.

Here, we will explain the importance of HGFs in the study of periodontitis. (1) HGFs are the most prominent cells in periodontal tissue. LPS-treated HGFs produce inflammatory chemical mediators, such as PGE${}_{2}$, and inflammatory cytokines such as IL-6 and IL-8. (2) More importantly, unlike macrophages, HGFs continue to produce PGE${}_{2}$ [26], IL-6, and IL-8 [27] in the presence of LPS. From these findings, the large amount of chemical mediators and cytokines derived from HGFs may be contained in periodontal tissues. Therefore, we believe that examining the effects of pharmaceuticals on HGFs is needed in the study of periodontitis.

### 3.2. Brief Summary of Arachidonic Acid Cascade

**Figure 2:**
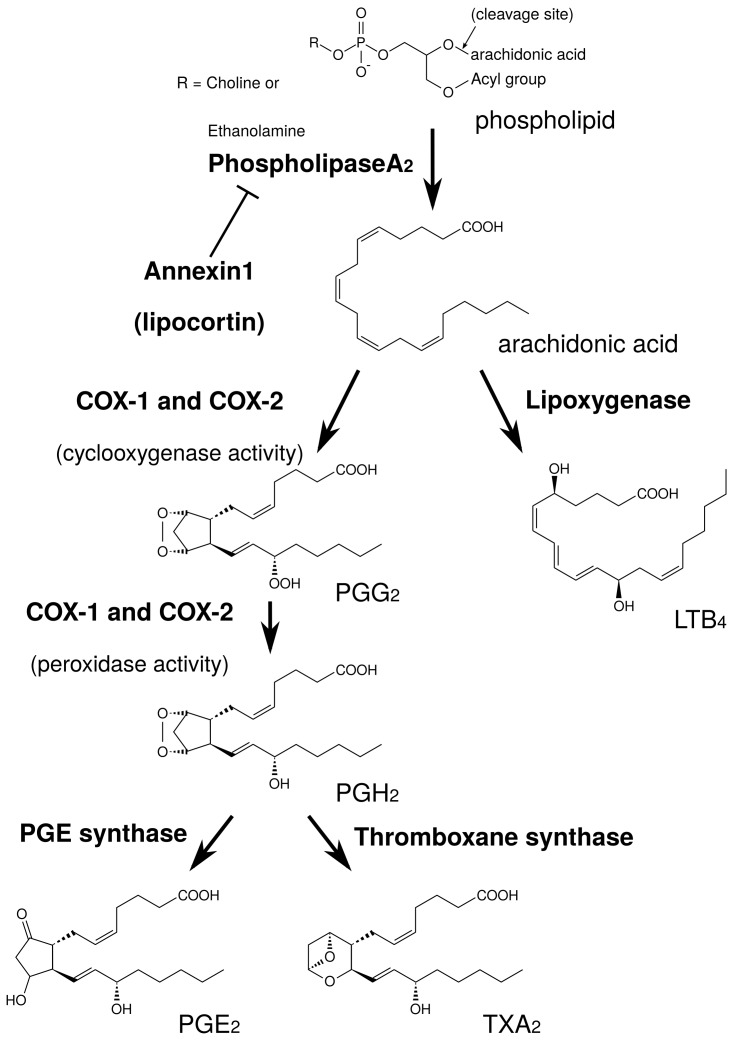
Simplified schema of arachidonic acid cascade.

At first, we explain arachidonic acid cascade briefly, focusing on sites of action for herbs and ingredients. PGE${}_{2}$ is produced by arachidonic acid cascade (Figure 2). Phospholipids in plasma membrane are digested by phospholipase A${}_{2}$ (PLA${}_{2}$), producing arachidonic acid. Cyclooxygenases (COXs) convert arachidonic acid into PGH${}_{2}$, and thereafter PGE synthase converts into PGE${}_{2}$.

PLA${}_{2}$ is the most upstream enzyme in the arachidonic acid cascade and releases arachidonic acid from the plasma membrane. PLA${}_{2}$ forms a superfamily and is classified into cytosolic PLA${}_{2}$ (cPLA${}_{2}$), calcium-independent PLA${}_{2}$ (iPLA${}_{2}$), secretory PLA${}_{2}$ (sPLA${}_{2}$), and others [28]. Among these isoforms, cPLA${}_{2}$ is the primary isoform in HGFs from the results using PLA${}_{2}$ inhibitors [29]. cPLA${}_{2}$ activity is directly regulated by extracellular signal-regulated kinase (ERK). The active form of ERK (phosphorylated ERK) phosphorylates Ser505 of cPLA${}_{2}$ and activates cPLA${}_{2}$ [30,31,32]. Therefore, the suppression of ERK phosphorylation leads to the suppression of cPLA${}_{2}$ activation and the reduction of PGE${}_{2}$ production [30,31,32]. In contrast, annexin1, also named as lipocortin, is an anti-inflammatory mediator produced by steroidal anti-inflammatory drugs (SAIDs) that inhibits PLA${}_{2}$ activity [33,34].

COX is classified into COX-1 and COX-2. COX-1 is constitutive expressed at low level, and is involved in normal functions such as protection of gastric mucosa. In contrast, COX-2 is induced by the various stimuli such as LPS and peptidoglycan, and involved in inflammatory response. The expression of COX-2 is upregulated by NF-κB. The reduction of PGE${}_{2}$ by anti-inflammatory drugs is one of the important mechanisms. Acid NSAIDs inhibit both COX-1 and COX-2 activities. The inhibition of COX-2 improve inflammatory response, while the inhibition of COX-1 causes gastric irritation. SAIDs also have powerful anti-inflammatory effects, and inhibit NF-κB activity and suppress COX-2 expression.

Recently, protein kinase A (PKA) pathway is reported to regulate LPS-induce PGE${}_{2}$ production in HGFs [35]. PKA inhibitor (H-89) reduced LPS-induced PGE${}_{2}$ production in a concentration-dependent manner. In contrast, PKA activator (dibutyryl cAMP; dbcAMP) and drugs which increased intracellular cAMP (adrenaline and aminophylline) increased LPS-induced PGE${}_{2}$ production in a concentration-dependent manner. However, the effects of PKA pathway on arachidonic acid cascade have not been examined in this report [35].

### 3.3. Effect of Herbal Medicines on Periosontal Disease

Similar to NSAIDs, several herbal medicines also reduce PGE${}_{2}$ production. Examples of herbal medicine which have been reported to reduce PGE${}_{2}$ production in in vitro and/or animal models are shown in Table 1. In particular, we reported that kakkonto (TJ-1), shosaikoto (TJ-9), hangeshashinto (TJ-14), shinbuto (TJ-30), ninjinto (TJ-32), and orento (TJ-120) reduced LPS-induced PGE${}_{2}$ production using HGFs [36,37,38,39,40]. Other groups have also demonstrated that several herbal medicines reduced PGE${}_{2}$ production using human periodontal ligament cells [41], human monocytes [42], mouse macrophage RAW264.7 cells [43,44], human oral keratinocytes [7], and animals [45,46,47,48].

We introduce briefly the effects and mechanisms of herbal medicines on periodontitis in clinical, animal, and/or in vitro studies. Moreover, in this section, we will demonstrate the effects of herbal medicines on the reduction of PGE${}_{2}$ in HGFs. From our data, the mechanisms of these herbal medicines on arachidonic acid cascade are divided into three groups as follows.

- Shosaikoto (TJ-9) inhibited COX-2 activity and suppressed COX-2 expression, but did not alter cPLA${}_{2}$ expression (the effects on annexin1 expression and ERK phosphorylation were not examined) [37]. Hangeshashinto (TJ-14) inhibited both COX-1 and COX-2 activities, and suppressed cPLA${}_{2}$ and COX-2 expressions and ERK phosphorylation [38]. Therefore, these herbal medicines are suggested to inhibit arachidonic acid cascade at multiple points.
- Shinbuto (TJ-30) and ninjinto (TJ-32) enhanced annexin1 expression, but did not alter ERK phosphorylation and COX activity [39]. However, the contribution of enhancement of annexin1 expression is considered to be small because shokyo, which is the main herb in shinbuto to reduce PGE${}_{2}$ production, did not affect annexin1 expression.
- Kakkonto (TJ-1) suppressed ERK phosphorylation, but neither inhibited COXs activities nor suppressed the expression of molecules in arachidonic acid cascade [36]. In addition, orento (TJ-120) suppressed ERK phosphorylation, but neither inhibited COXs activities nor suppressed the expression of molecules in arachidonic acid cascade, but rather increased COX-2 expression [40]. However, its contribution in the suppression of ERK phosphorylation is considered to be small as described at keihi (*Cinnamomi Cortex*). Indeed, we did not examine the direct effect of herbal medicines on cPLA${}_{2}$ activity. Nevertheless, we consider that these herbal medicines inhibit cPLA${}_{2}$ activity and that this effect is due to shokyo (*Zingiberis Rhizoma*) and kankyo (*Zingiberis Processum Rhizoma*) as described below.

### 3.4. Effect of Herbs on Arachidonic Acid Cascade

**Figure 3:**
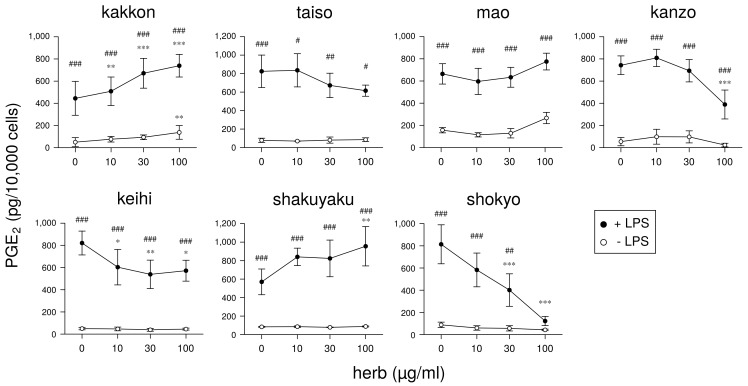
The effect of herbs in kakkonto (TJ-1) on PGE${}_{2}$ production: This figure is cited from Ara and Sogawa [29] (CC-BY-4.0) and modified for this review.

**Figure 4:**
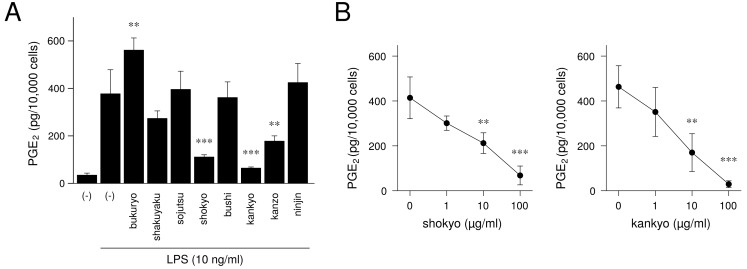
The effect of herbs in shinbuto (TJ-30) and ninjinto (TJ-32) on PGE${}_{2}$ production: This figure is cited from Ara and Sogawa [39] (CC-BY-4.0) and modified for this review. (**A**): Effect of each herb, (**B**): Concentration-dependent effects of shokyo and kankyo.

Next, we will demonstrate the experimental results at the herb level. The ingredients in the formula of herbal medicines that were used are shown in Table 2, Table 3, Table 4, Table 5, Table 6 and Table 7. In our experiments at the herb level, shokyo (*Zingiberis Rhizoma*), kankyo (*Zingiberis Processum Rhizoma*), kanzo (*Glycyrrhizae Radix*), and keihi (*Cinnamomi Cortex*) reduced PGE${}_{2}$ production (Figure 3 and Figure 4) [29,39]. We summarized major ingredients in herbs and their mechanism against arachidonic acid cascade in Table 8. In addition to these four herbs, ogon (*Scutellariae Radix*), and oren (*Coptidis Rhizoma*) are shown in Table 8 because ogon (included in shosaikoto and hangeshashinto) and oren (included in hangeshashinto and orento) also have several bioactive ingredients such as flavonoids, saponin, and chalcones. We will describe the effects and mechanisms of these herbs, particularly shokyo and kankyo, on arachidonic cascade.

#### 3.4.1. Shokyo (*Zingiberis Rhizoma*)/Kankyo (*Zingiberis Processum Rhizoma*)

Shokyo is the powdered rhizome of *Zingiber offinale* Roscoe (ginger), and kankyo is the steamed and powdered rhizome of ginger. Both shokyo and kankyo are the aqueous extracts of ginger. Among the herbal medicines shown in Table 1, shokyo is included in kakkonto (TJ-1), shosaikoto (TJ-9), shinbuto (TJ-30), saireito (TJ-114), and orento (TJ-120), and kankyo is included in hangeshashinto (TJ-14) and ninjinto (TJ-32). Many reports have demonstrated that ginger possesses anti-inflammatory effects in human [80,81] and animal models [82,83,84], and in vitro [85]. Ginger has been widely used in diet and as a treatment for rheumatoid arthritis, fever, emesis, nausea, and migraine headache [80]. A recent systematic review shows that the extracts of ginger including turmeric, ginger, Javanese ginger, and galangal are clinically effective as hypoanalgesic agents [81]. In an animal model, the aqueous extract of ginger significantly reduced serum PGE${}_{2}$ level by oral or intraperitoneal administration in rats [82]. Moreover, crude hydroalcoholic extract of ginger reduced the serum level of PGE${}_{2}$, and improved tracheal hyperreactivity and lung inflammation induced by LPS in rats [83]. Ethanol extract of ginger reduced the tissue level of PGE${}_{2}$, and improved acetic acid-induced ulcerative colitis in rats [84].

**Figure 5:**
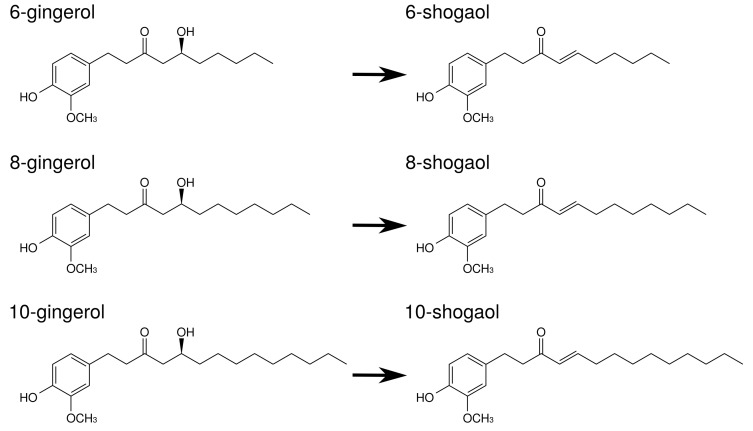
The structures of ingredients in shokyo (*Zingiberis Rhizoma*) and kankyo (*Zingiberis Processum Rhizoma*).

Gingerols and shogaols are the major ingredients in ginger. Their structures are indicated in Figure 5. With prolonged storage or heat-treatment of ginger, gingerols are converted to shogaols, which are the dehydrated form of the gingerols [80] (Figure 5). Therefore, kankyo contains a larger amount of shogaols than shokyo although both shokyo and kankyo contain gingerols and shogaols. In in vitro models, gingerols and shogaols have been reported to reduce PGE${}_{2}$ production by several mechanisms. The effects of gingerols and shogaols on arachidonic acid cascade are briefly summarized in Table 8.

1. Gingerols and shogaols inhibit COX-2 activity. Their IC${}_{50}$ values were μM order: 6-gingerol (>50 μM), 8-gingerol (10.0 μM), 10-gingerol (3.7 μM), 6-shogaol (2.1 μM), and 8-shogaol (7.2 μM) in human lung adenocarcinoma A549 cells [49], and 10-gingerol (32.0 μM), 8-shogaol (17.5 μM), 10-shogaol (7.5 μM) in a cell-free assay [50].
2. Gingerols and shogaols suppress COX-2 expression. For example, 6-, 8-, and 10-gingerol suppressed COX-2 expression in LPS-treated human leukemic monocyte lymphoma U937 cells [51]. Similarly, 6-gingerol and 6-shogaol suppressed LPS-induced COX-2 expression in mouse macrophage RAW264.7 cells [52], mouse microglial BV-2 cells [53], and primary rat astrocytes [86]. 6-Gingerol suppressed COX-2 expression in TPA-treated mouse skin in vivo [54].
3. As aforementioned, the expression of COX-2 is regulated by NF-κB. Gingerols and shogaols are reported to suppress NF-κB activation, and to downregulate COX-2 expression. For example, 6-shogaol suppressed LPS-induced NF-κB activation in RAW264.7 cells [52], mouse primary cultured microglia cells [53], and human breast cancer MDA-MB-231 cells [56]. 6-Shogaol suppressed TPA-induced NF-κB activation in mouse skin [54]. Similarly, 6-gingerol suppressed *Vibrio cholerae*-induced NF-κB activation in human intestinal epithelial cells [55]. These results suggest that gingerols and shogaols suppress NF-κB activation directly or indirectly, leading to the inhibition of COX-2 expression.
4. Gingerols and shogaols inhibit PLA${}_{2}$ activities [57]. In more detail, iPLA${}_{2}$ activity was inhibited by 6-, 8-, and 10-gingerol and 6-, 8-, and 10-shogaol, whereas cPLA${}_{2}$ activity was inhibited by 6-gingerol and 6-, 8-, and 10-shogaol. In particular, IC${}_{50}$ values of 10-shogaol against iPLA${}_{2}$ and cPLA${}_{2}$ were 0.7 μM and 3 μM, respectively, in U937 cells.

As aforementioned, many reports have examined the effects of ginger. However, there is little report using ginger as “shokyo” and “kankyo.” For this reason, we examined the mechanism of the actions of shokyo and kankyo on the reduction of PGE${}_{2}$ production in HGFs. Shokyo and kankyo concentration-dependently reduced LPS-induced PGE${}_{2}$ production by HGFs, and the effects of kankyo were slightly stronger than those of shokyo (Figure 4) [39]. The effects of shokyo and kankyo on arachidonic cascade in HGFs are described as follows.

- Both shokyo and kankyo only slightly increased cPLA${}_{2}$ expression, and did not alter annexin1 expression [39].
- Shokyo did not alter LPS-induced ERK phosphorylation in HGFs [29] (but we have not examined the effect of kankyo). Therefore, shokyo (and perhaps kankyo) may have little to no effect on cPLA${}_{2}$ activation, and the subsequent arachidonic acid production.
- Both shokyo and kankyo did not inhibit COX-2 and PGE synthase activities, and did not alter LPS-induced COX-2 expression in HGFs [29,39]. These findings suggest shokyo and kankyo primarily inhibit cPLA${}_{2}$ activity in HGFs. Although we have no direct data to show that shokyo and kankyo inhibit cPLA${}_{2}$ activity, this assumption is consistent with the results that ginger (and gingerols/shogaols) inhibits both iPLA${}_{2}$ and cPLA${}_{2}$ activities [57].

As described above, our data that shokyo did not alter COX-2 activity and COX-2 expression are different from those of gingerols and shogaols in Table 8. Although there is no obvious evidence, the reason may be the preparation method of shokyo and kankyo. Gingerols and shogaols are extremely hydrophobic by their structures. These ingredients were extracted from hydrophobic phase, whereas shokyo and kankyo were prepared by decoction. Therefore, hydrophobic ingredients such as gingerol and shogaol are unlikely to be extracted, and their concentration might be lower than those in previous reports. Quantification of these ingredients is needed to explain these discrepancies.

#### 3.4.2. Kanzo (*Glycyrrhizae Radix*)

Kanzo is the powdered root or stolon of *Glycyrrhiza uralensis* Fischer (licorice). Among the herbal medicines shown in Table 1, kanzo is included in kakkonto (TJ-1), shosaikoto (TJ-9), hangeshashinto (TJ-14), ninjinto (TJ-32), rikkosan (TJ-110), saireito (TJ-114), and orento (TJ-120). Licorice is also known to have anti-inflammatory effects [87] such as inhibition of COX-2 activity [46].

**Figure 6:**
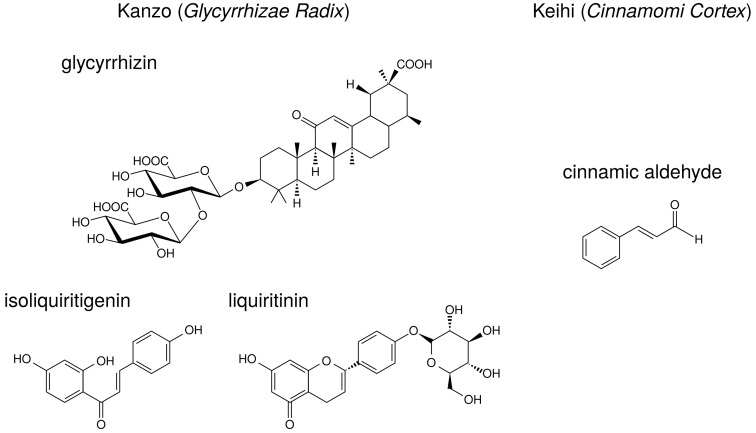
The structures of ingredients in kanzo (*Glycyrrhizae Radix*) and keihi (*Cinnamomi Cortex*).

Licorice contains triperpene saponin such as glycyrrhizin (glycyrrhizin acid), and chalcones such as liquiritin and isoliquiritigenin. Their structures are indicated in Figure 6. Glycyrrhizin, liquiritin, and isoliquiritigenin are reported to reduce PGE${}_{2}$ production. The effects of these ingredients on arachidonic acid cascade are briefly summarized in Table 8.

1. Glycyrrhizin suppressed COX-2 expression in LPS-treated mouse microglial BV2 cells [58] and uterus of ovariectominezed mice [59]. Moreover, orally administrated glycyrrhizin suppressed COX-2 expression in the cerebral cortex of LPS-treated mice [60]. Liquiritin and isoliquiritigenin also suppressed LPS-induced COX-2 expression in RAW264.7 cells [63] and BV2 cells [58].
2. Glycyrrhizin suppressed TNF-α or IL-1β-induced NF-κB activation in human lung epithelial A549 cells [61]. Isoliquiritigenin also suppressed NF-κB activity and suppressed LPS-induced COX-2 expression in RAW264.7 cells [64].
3. Glycyrrhizin and isoliquiritigenin inhibited TLR4 (receptor of LPS) homodimerization and downstream signal pathway [62], resulting in the suppression of COX-2 expression.

Indeed, although glycyrrhizin has anti-inflammatory effects, glycyrrhizin is known to show a serious adverse effect, pseudohyperaldosteronism. Excessive dietary intake of licorice can cause a syndrome mimicking hypermineralocorticoidism, characterized by hypertension, hypokalemia, alkalosis, and reduced plasma renin [88,89,90,91]. Glycyrrhizin inhibits 11β-hydroxysteroid dehydrogenase type 2 (11β-HSD2), which converts active glucocorticoid cortisol to inactive cortisone [92]. This inhibition results in the activation of renal mineralocorticoid receptors by cortisol, inducing Na${}^{+}$ reabsorption, K${}^{+}$ excretion, hypertension, hypokalemia, and metabolic alkalosis. These phenotypes are similar to that of apparent mineralocorticoid excess syndrome. [91,93].

We examined the mechanism of the action of kanzo on the reduction of PGE${}_{2}$ production in HGFs. However, the effects of kanzo on arachidonic acid cascade in HGFs cannot be explained by those of glycyrrhizin, liquiritin, and isoliquiritigenin.

- As reported previously [46], kanzo inhibited COX-2 activity because kanzo decreased LPS-induced PGE${}_{2}$ production when arachidonic acid was added [29]. In contrast, kanzo did not inhibit PGE synthase activity because kanzo did not alter LPS-induced PGE${}_{2}$ production when PGH${}_{2}$ was added [29].
- Kanzo increased both cPLA${}_{2}$ and annexin1 expressions [29], thus leaving the effect of kanzo on PLA${}_{2}$ unconcluded.
- Kanzo increased LPS-induced COX-2 expression [29] although glycyrrhizin, liquiritin, and isoliquiritigenin suppressed COX-2 expression [58,59,60,63,64].
  This result is the same as those observed using orento [40] and saireito [44], which contain kanzo.

Therefore, these effects of kanzo were different from those of glycyrrhizin, liquiritin, and isoliquiritigenin, suggesting that other ingredients may contribute to our findings. In addition, not all herbal medicines which contain kanzo increased annexin1 as kakkonto, hangeshashinto, and orento did not alter annexin1 expression.

#### 3.4.3. Keihi (*Cinnamomi Cortex*)

Keihi is the powdered bark of *Cinnamomum cassia* (cinnamon). Among the herbal medicines shown in Table 1, keihi is included in kakkonto (TJ-1), saireito (TJ-114), and orento (TJ-120). Cinnamon has been widely used for the treatment of fever and inflammation [28]. Cinnamon improves nephritis, purulent dermatitis, and hypertension, and it also enhances wound healing. Cinnamon extracts have been used for the improvement or prevention of common cold, diarrhea, and pain [28]. Ethanol-extract of *C. cassia* reduced LPS-induced PGE${}_{2}$ production by RAW264.7 cells, and it suppressed NF-κB activity and the following COX-2 expression [66].

Keihi contains the ingredients such as cinnamic aldehyde, cinnamic alcohol, cinnamic acid, and coumarin. The structure of cinnamic aldehyde is indicated in Figure 6. Cinnamic aldehyde is reported to reduce PGE${}_{2}$ production. The effects of cinnamic aldehyde on arachidonic acid cascade are briefly summarized in Table 8.

1. Cinnamic aldehyde suppressed carrageenan-induced COX-2 expression and improved footpad edema in mice [65]. Cinnamic aldehyde, but not others, suppressed LPS-induced COX-2 expression and decreased PGE${}_{2}$ production by RAW264.7 cells [65,66].
2. Cinnamic aldehyde suppressed LPS-induced NF-κB activity in RAW264.7 cells and TLR4- expressing HEK293 cells [67].
3. Cinnamic aldehyde inhibited IL-1β-induced COX-2 activity in rat cerebral microvascular endothelial cells although its effect is weak [68].
4. Cinnamic aldehyde inhibited TLR4 oligomerization and downstream signal pathway, which include NF-κB. Sulfhydryl modification is suggested to be an important contributing factor for the regulation of TLR4 activation [69].

We examined the mechanism of action of keihi on the reduction of PGE${}_{2}$ production in HGFs. However, the effects of keihi on arachidonic acid cascade in HGFs cannot be explained by that of cinnamic aldehyde.

- Keihi inhibited COX-2 activity because keihi decreased LPS-induced PGE${}_{2}$ production when arachidonic acid is added [29]. This mechanism is accounted for by that of cinnamic aldehyde. In contrast, keihi did not inhibit PGE synthase activity as well as kanzo.
- As well as kakkonto [81] and orento [40], keihi suppressed ERK phosphorylation in LPS-treated HGFs [29], leading to inhibit cPLA${}_{2}$ activation. However, the contribution of suppression of ERK phosphorylation is considered to be small because the ability of keihi to decrease LPS-induced PGE${}_{2}$ production was weak (Figure 3).
- Keihi increased LPS-induced COX-2 expression.

Therefore, these effects of keihi are different from that of cinnamic aldehyde, suggesting that other ingredients may contribute to our findings.

#### 3.4.4. Ogon (*Scutellariae Radix*)

Ogon is the powdered root of *Scutellaria baicalensis* Georgi. Among the herbal medicines shown in Table 1, ogon is included in shosaikoto (TJ-9), hangeshashinto (TJ-14), and saireito (TJ-114). Among the herbs constituting saireito, ogon is reported to reduce PGE${}_{2}$ production by LPS-treated RAW264.7 cells [44].

**Figure 7:**
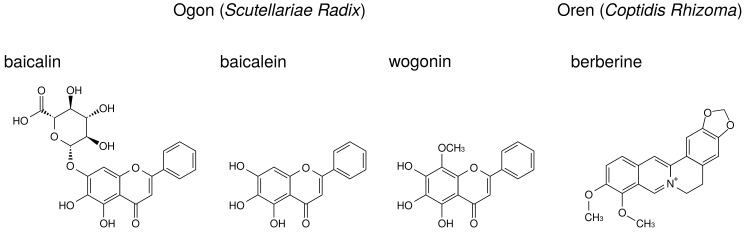
The structures of ingredients in ogon (*Scutellariae Radix*) and oren (*Coptidis Rhizoma*).

The major ingredients of ogon are flavonoids such as baicalin, baicalein, and wogonin. Their structures are indicated in Figure 7. Baicalin is the glucuronide of baicalein and is an inactive form. Administered baicalein is metabolized to baicalin, which is an active form. Baicalin, baicalein, and wogonin reduce PGE${}_{2}$ production in human oral keratinocytes [7] and RAW264.7 cells [94].

1. Wogonin suppressed LPS-induced COX-2 expression in RAW264.7 cells [73,74], whereas baicalin and baicalein did not [73]. Other group demonstrated that baicalein (but not baicalin) suppressed LPS-induced COX-2 expression in RAW264.7 cells [71]. This discrepancy may be due to the concentrations of LPS and flavonoids among these reports. Moreover, baicalein and wogonin suppressed COX-2 expression in human oral keratinocytes [7].
2. Baicalin [70], baicalein [72], and wogonin [7] suppressed NF-κB activity.
3. Baicalin, baicalein, and wogonin did not inhibit COX-2 activity in RAW264.7 cells [73].

Our data indicate that shosaikoto and hangeshashinto, which include ogon, suppressed LPS- induced COX-2 expression in HGFs [37,38]. This mechanism is accounted for by those of baicalin, baicalein, and wogonin.

#### 3.4.5. Oren (*Coptidis Rhizoma*)

Oren is the powdered rhizome of *Coptis japonica* Makino, *Coptis chinensis* Franchet, *Coptis deltoidea* C. Y. Cheng et Hsiao, or *Coptis teeta* Wallich (Ranunculaceae). Among the herbal medicines shown in Table 1, oren is included in hangeshashinto (TJ-14) and orento (TJ-120).

Berberine, one of benzylisoquinoline alkaloid, is the major ingredient of oren. The structure of berberine is indicated in Figure 7. Berberine is reported to reduce PGE${}_{2}$ production. The effects of berberine on arachidonic acid cascade are briefly summarized in Table 8.

1. Berberine suppressed NF-κB activation and COX-2 expression in human leukemia Jurkat cells [75] and oral cancer OC2 and KB cells [95,96].
2. Berberine suppressed MAP kinases phosphorylation (including ERK) and activated AMP-activated protein kinase (AMPK) in peritoneal macrophages and RAW 264.7 cells [76], BV-2 cells [77], and melanoma cells [78]. Therefore, berberine is considered to inhibit cPLA${}_{2}$ activation through suppression of ERK phosphorylation. In addition, because AMPK is reported to suppress NF-κB activation [97], berberine suppressed COX-2 expression due to activation of AMPK.

### 3.5. Conclusion about Herbal Medicines and Herbs

We have described the effects of herbal medicines, herbs, and their ingredients on arachidonic acid cascade in this review. Several herbal medicines show reduced LPS-induced PGE${}_{2}$ production by HGFs. These results suggest that these herbal medicines may be effective in the improvement of the inflammatory symptoms in periodontitis. Herbal medicines must be properly selected by the patterns of each patient —excess patterns, medium patterns, or deficiency patterns. Among the herbal medicines in our studies, kakkonto (TJ-1) and orento (TJ-120) are used for the patients with excess patterns. Shosaikoto (TJ-9), hangeshashinto (TJ-14), and orento are used for the patients with medium patterns. Shinbuto (TJ-30) and ninjinto (TJ-32) are used for the patients with deficiency patterns. Therefore, it may be possible to use appropriate herbal medicines to patients with any pattern.

As shown in the above-mentioned descriptions, not all effects of herbal medicines are explainable by the effects of herbs constituting herbal medicines. Similarly, not all effects of herbs are explainable by the effects of ingredients contained in herbs. Experiments using “herbal medicines” or “herbs” themselves may be important rather than those using ingredients. The concentrations of these hydrophobic ingredients may also be low because the herbs that we used are water-soluble fractions. Therefore, it is considered that the concentrations of their ingredients need to be measured. Moreover, the unanalyzed ingredients other than those explained in this review are likely to be present. It is to be desired that further analyses reveal the novel ingredients and their action of mechanisms.

## 4. Anti-Osteoclastogenic Effects of Natural Products

Like periodontitis (PD), rheumatoid arthritis (RA) is a disease associated with inflammation and bone destruction. Although therapeutics of RA have recently advanced with the development of antibody drugs, natural substances displaying anti-inflammatory and anti-osteoclast characteristics against RA are still being used as widely as they have been in the past.

Some studies have revealed a relationship between PD and RA. RA prevalence is increased in patients with PD [98,99]. The presence of PD may contribute to the progression of RA; that is, RA patients with PD receiving non-surgical periodontal treatment resulted in a noteworthy improvement in the clinical outcome for RA [100]. From the aspect of the clinical marker, RA and PD are similar in cytokines and mediators involved in inflammation and bone destruction [101]. For example, TNF-α, receptor activator of nuclear factor-κB ligand (RANKL), and matrix metalloproteinase (MMP) family increase in production in RA and PD [102,103,104,105,106]. Due to these similarities, natural products used for RA are probably effective for PD.

**Figure 8:**
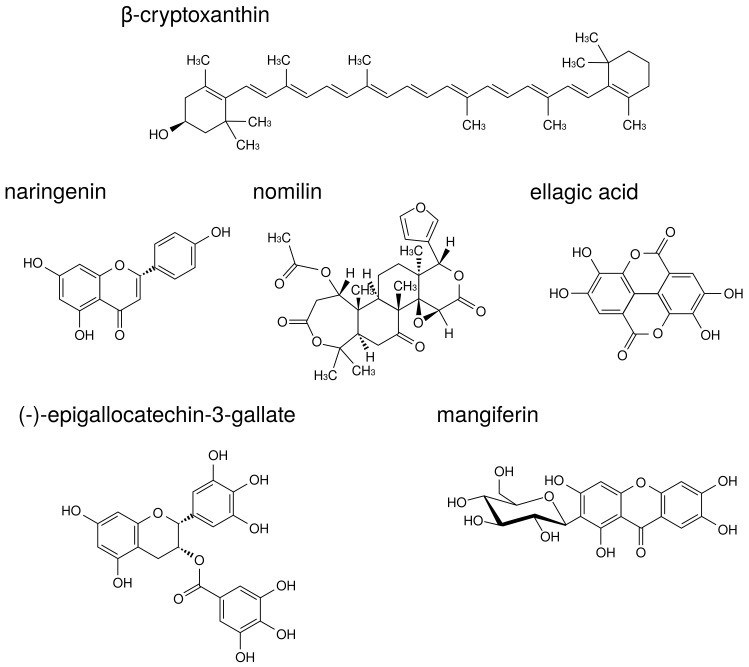
The structures of natural products.

The structures of natural products described in this review are indicated in Figure 8.

Epidemiological studies have revealed a positive correlation between bone health and increased consumption of fruits and vegetables [107,108]. Some fruits and vegetables contain components that inhibit both inflammation and osteoclast activity.

β-Cryptoxanthin is a carotenoid present in a wide range of citrus fruits and in *Diospyros kaki* Thunb., *Physalis alkekengi* L., etc. β-Cryptoxanthin has a potent inhibitory effect on osteoclast-like cell formation in mouse marrow culture [109]. Moreover, in a mouse model of PD, β-cryptoxanthin suppressed bone resorption in the mandibular alveolar bone in vitro and restored alveolar bone loss induced by LPS in vivo [110].

Naringenin is a flavonoid contained in citrus fruits such as oranges and grapefruits. Accumulating evidence has suggested that naringenin modulates chronic inflammation [111]. In a murine model of collagen-induced arthritis, naringenin inhibited pro-inflammatory cytokine production by decreasing MAPK and NF-κB signaling activation [112]. La et al. showed naringenin thus holds promise as a therapeutic and preventive agent for bone-related diseases such as PD [113]. Thus, there are cases in which components demonstrating anti-osteoclast behavior are demonstrated to be effective against PD. In addition to naringenin, citrus fruits contain components that suppress osteoclast activity via MAPK. Nomilin, a limonoid present in citrus fruits, displays inhibitory effects on osteoclastic differentiation through the suppression of MAPK signaling pathways [114].

Ellagic acid is a polyphenol contained in berries, pomegranates, nuts, etc. Ellagic acid has an anti-inflammatory effect in various organs such as the liver, stomach, small intestine, and skin [115,116,117,118]. Moreover, ellagic acid has anti-osteoclast activity and significantly reduced serum levels of pro-inflammatory cytokines, TNF-α, IL-1β, and IL-17 in RA model mice [119]. A recent study supported the traditional use of *Geum urbanum* L. root contained ellagic acid derivatives in cavity inflammation including mucositis, gingivitis, and PD [120].

Additional useful components against both RA and PD have been found in tea. (-)-Epigallocatechin-3-gallate (EGCG) is a major catechin derivative present in green tea. Previous studies have also suggested that EGCG decreases MMP-1, MMP-2, and MMP-3 production by RA synovial fibroblasts, thereby preventing further cartilage and bone destruction [121,122]. Moreover, it has been reported that EGCG selectively inhibited IL-1β-induced IL-6 synthesis in RA synovial fibroblasts and suppressed IL-6 trans-signaling via upregulation of an endogenous inhibitor, a soluble gp130 [123]. Clinical study of EGCG suggested that local drug delivery utilizing green tea extract could be used as an adjunct in the treatment of chronic PD [124].

Traditional medicine in Ayurveda also presents useful teas against RA and PD. *Salacia reticulata* Wight is a plant native to Sri Lanka that has been used for the prevention of RA, gonorrhea, and skin disease. We previously reported that leaf of *S. reticulata* alleviates collagen antibody-induced arthritis in RA model mice [125]. *S. reticulata* contains a polyphenol known as mangiferin that inhibits osteoclastic bone resorption by promoting ERβ mRNA expression in mouse bone marrow macrophage cells [126].

In conclusion, natural products displaying both anti-inflammation and anti-osteoclast characteristics are suggested to be useful for the prevention and treatment of PD. 

## Figures and Tables

**Table 1:** Japanese traditional herbal medicines which are reported to reduce PGE${}_{2}$ production.

Herbal Medicine	Cells or Animal	References
kakkonto (TJ-1)	HGFs	[36]
shosaikoto (TJ-9)	HGFs	[37]
	human monocytes	[42]
	mouse	[45]
hangeshashinto (TJ-14)	HGFs	[38,41]
	human periodontal ligament cells	[41]
	human oral keratinocytes	[7]
	rat	[46,47,48]
shinbuto (TJ-30)	HGFs	[39]
ninjinto (TJ-32)	HGFs	[39]
rikkosan (TJ-110)	RAW264.7	[43]
saireito (TJ-114)	RAW264.7	[44]
orento (TJ-120)	HGFs	[40]

**Table 2:** The ingredients in the kakkonto (TJ-1) formula.

Japanese Name	Scientific Name	Amount	Amount *
(Latin Name)		(g)	(g/g of Product)
kakkon	*Pueraria lobata* Ohwi	4.0	0.111
(*Puerariae Radix*)			
taiso	*Ziziphus jujuba* Miller var. *inermis* Rehder	3.0	0.083
(*Zizyphi Fructus*)			
mao	*Ephedra sinica* Stapf	3.0	0.083
(*Ephedrae Herba*)	*Ephedra intermedia* Schrenk et C.A.Meyer		
	*Ephedra equisetina* Bunge		
kanzo	*Glycyrrhiza uralensis* Fischer	2.0	0.056
(*Glycyrrhizae Radix*)	*Glycyrrhiza glabra* Linné		
keihi	*Cinnamomum cassia* Blume	2.0	0.056
(*Cinnamomi Cortex*)			
shyakuyaku	*Paeonia lactiflora* Pallas	2.0	0.056
(*Paeoniae Radix*)			
shokyo	*Zingiber officinale* Roscoe	2.0	0.056
(*Zingiberis Rhizoma*)			
total		18.0	0.500

* 7.5 g of kakkonto product contains 3.75 g of a dried extract of the mixed crude drugs.

**Table 3:** The ingredients in the shosaikoto (TJ-9) formula.

Japanese Name	Scientific Name	Amount	Amount *
(Latin Name)		(g)	(g/g of Product)
saiko	*Bupleurum falcatum* Linné	7.0	0.175
(*Bupleuri Radix*)			
hange	*Pinellia ternata* Breitenbach	5.0	0.125
(*Pinelliae tuber*)			
ogon	*Scutellaria baicalensis* Georgi	3.0	0.075
(*Scutellariae radix*)			
taiso	*Ziziphus jujuba* Miller var. *inermis* Rehder	3.0	0.075
(*Zizyphi Fructus*)			
ninjin	*Panax ginseng* C.A. Meyer	3.0	0.075
(*Ginseng Radix*)			
kanzo	*Glycyrrhiza uralensis* Fischer	2.0	0.050
(*Glycyrrhizae Radix*)	*Glycyrrhiza glabra* Linné		
shokyo	*Zingiber officinale* Roscoe	1.0	0.025
(*Zingiberis Rhizoma*)			
total		24.0	0.600

* 7.5 g of shosaikoto product contains 4.5 g of a dried extract of the mixed crude drugs.

**Table 4:** The ingredients in the hangeshashinto (TJ-14) formula.

Japanese Name	Scientific Name	Amount	Amount *
(Latin Name)		(g)	(g/g of Product)
hange	*Pinellia ternata* Breitenbach	5.0	0.162
(*Pinelliae tuber*)			
ogon	*Scutellaria baicalensis* Georgi	2.5	0.081
(*Scutellariae radix*)			
kankyo	*Zingiber officinale* Roscoe	2.5	0.081
(*Zingiberis Processum Rhizoma*)			
kanzo	*Glycyrrhiza uralensis* Fischer	2.5	0.081
(*Glycyrrhizae Radix*)	*Glycyrrhiza glabra* Linné		
taiso	*Ziziphus jujuba* Miller var. *inermis* Rehder	2.5	0.081
(*Zizyphi Fructus*)			
ninjin	*Panax ginseng* C.A. Meyer	2.5	0.081
(*Ginseng Radix*)			
oren	*Coptis japonica* Makino	1.0	0.032
(*Coptidis rhizoma*)	*Coptis chinensis* Franchet		
	*Coptis deltoidea* C. Y. Cheng et Hsiao		
	*Coptis teeta* Wallich		
total		18.5	0.600

* 7.5 g of hangeshashinto product contains 4.5 g of a dried extract of the mixed crude drugs.

**Table 5:** The ingredients in the shinbuto (TJ-30) formula.

Japanese Name	Scientific Name	Amount	Amount *
(Latin Name)		(g)	(g/g of Product)
bukuryo	*Wolfiporia cocos* Ryvarden et Gilbertson	4.0	0.089
(*Poria Sclerotium*)	(*Poria cocos* Wolf)		
shakuyaku	*Paeonia lactiflora* Pallas	3.0	0.067
(*Paeoniae Radix*)			
sojutsu	*Atractylodes lancea* De Candolle	3.0	0.067
(*Atractylodis Lanceae Rhizoma*)	*Atractylodes schinensis* Koidzumi		
shokyo	*Zingiber officinale* Roscoe	1.5	0.033
(*Zingiberis Rhizoma*)			
bushi	*Aconitum carmichaeli* Debeaux	0.5	0.011
(*Processi Aconiti Radix*)	*Aconitum japonicum* Thunberg		
total		12.0	0.267

* 7.5 g of shinbuto product contains 2.0 g of a dried extract of the mixed crude drugs.

**Table 6:** The ingredients in the ninjinto (TJ-32) formula.

Japanese Name	Scientific Name	Amount	Amount *
(Latin Name)		(g)	(g/g of Product)
kankyo	*Zingiber officinale* Roscoe	3.0	0.083
(*Zingiberis Processum Rhizoma*)			
kanzo	*Glycyrrhiza uralensis* Fischer	3.0	0.083
(*Glycyrrhizae Radix*)	*Glycyrrhiza glabra* Linné		
sojutsu	*Atractylodes lancea* De Candolle	3.0	0.083
(*Atractylodis Lanceae Rhizoma*)	*Atractylodes schinensis* Koidzumi		
ninjin	*Panax ginseng* C.A. Meyer	3.0	0.083
(*Ginseng Radix*)			
total		12.0	0.333

* 7.5 g of ninjinto product contains 2.5 g of a dried extract of the mixed crude drugs.

**Table 7:** The ingredients in the orento (TJ-120) formula.

Japanese Name	Scientific Name	Amount	Amount *
(Latin Name)		(g)	(g/g of Product)
hange	*Pinellia ternata* Breitenbach	6.0	0.133
(*Pinelliae tuber*)			
oren	*Coptis japonica* Makino	3.0	0.067
(*Coptidis rhizoma*)	*Coptis chinensis* Franchet		
	*Coptis deltoidea* C. Y. Cheng et Hsiao		
	*Coptis teeta* Wallich		
kankyo	*Zingiber officinale* Roscoe	3.0	0.067
(*Zingiberis Processum Rhizoma*)			
kanzo	*Glycyrrhiza uralensis* Fischer	3.0	0.067
(*Glycyrrhizae Radix*)	*Glycyrrhiza glabra* Linné		
keihi	*Cinnamomum cassia* Blume	3.0	0.067
(*Cinnamomi Cortex*)			
taiso	*Ziziphus jujuba* Miller var. *inermis* Rehder	3.0	0.067
(*Zizyphi Fructus*)			
ninjin	*Panax ginseng* C.A. Meyer	3.0	0.067
(*Ginseng Radix*)			
total		24.0	0.533

* 7.5 g of orento product contains 4.0 g of a dried extract of the mixed crude drugs.

**Table 8:** Major ingredients in herbs and their mechanism against arachidonic acid cascade.

Herb	Ingredients	Mechanisms	References
shokyo/kankyo	gingerol, shogaol	inhibition of COX-2 activity	[49,50]
		suppression of COX-2 expression	[7,51,52,53,54]
		suppression of NF-κB activation	[52,53,54,55,56]
		inhibition of PLA${}_{2}$ activity	[57]
kanzo	glycyrrhizin	suppression of COX-2 expression	[58,59,60]
		suppression of NF-κB activation	[61]
		inhibition of TLR4 homodimerization	[62]
	isoliquiritigenin	suppression of COX-2 expression	[58,63,64]
		suppression of NF-κB activation	[64]
		inhibition of TLR4 homodimerization	[62]
	liquiritin	suppression of COX-2 expression	[58]
keihi	cinnamic aldehyde	suppression of COX-2 expression	[65,66]
		suppression of NF-κB activation	[67]
		inhibition of COX-activity	[68]
		inhibition of TLR4 oligomerization	[69]
ogon	baicalin	suppression of COX-2 expression	[7,70]
		suppression of NF-κB activation	[70]
	baicalein	suppression of COX-2 expression	[7,71]
		suppression of NF-κB activation	[72]
	wogonin	suppression of COX-2 expression	[7,73,74]
		suppression of MAPK ^(a)^ phosphorylation	[7]
oren	berberin	suppression of COX-2 expression	[75]
		suppression of NF-κB activation	[75]
		suppression of MAPK ^(a)^ phosphorylation	[76,77,78,79]
		enhancement of AMPK ^(b)^	[76,77,78]

^(a)^ MAP kinases; ^(b)^ AMP-activated protein kinase.

## References

[1] Veilleux M., Moriyama S., Yoshioka M., Hinode D., Grenier D. (2018). A Review of Evidence for a Therapeutic Application of Traditional Japanese Kampo Medicine for Oral Diseases/Disorders. Medicines.

[2] Wang P. (2012). Kampo medicines for oral disease. Oral Ther. Pharmacol..

[3] Kono T., Satomi M., Chisato N., Ebisawa Y., Suno M., Asama T., Karasaki H., Matsubara K., Furukawa H. (2010). Topical Application of Hangeshashinto (TJ-14) in the Treatment of Chemotherapy-Induced Oral Mucositis. World J. Oncol..

[4] Aoyama T., Nishikawa K., Takiguchi N., Tanabe K., Imano M., Fukushima R., Sakamoto J., Oba M., Morita S., Kono T. (2014). Double-blind, placebo-controlled, randomized phase II study of TJ-14 (hangeshashinto) for gastric cancer chemotherapy-induced oral mucositis. Cancer Chemother. Pharmacol..

[5] Yamashita T., Araki K., Tomifuji M., Kamide D., Tanaka Y., Shiotani A. (2015). A traditional Japanese medicine– Hangeshashinto (TJ-14)–alleviates chemoradiation-induced mucositis and improves rates of treatment completion. Support Care Cancer.

[6] Kamide D., Yamashita T., Araki K., Tomifuji M., Shiotani A. (2017). Hangeshashinto (TJ-14) prevents radiation- induced mucositis by suppressing cyclooxygenase-2 expression and chemotaxis of inflammatory cells. Clin. Transl. Oncol..

[7] Kono T., Kaneko A., Matsumoto C., Miyagi C., Ohbuchi K., Mizuhara Y., Miyano K., Uezono Y. (2014). Multitargeted effects of hangeshashinto for treatment of chemotherapy-induced oral mucositis on inducible prostaglandin E2 production in human oral keratinocytes. Integr. Cancer Ther..

[8] Furusawa S., Wu J. (2007). The effects of biscoclaurine alkaloid cepharanthine on mammalian cells: Implications for cancer, shock, and inflammatory diseases. Life Sci..

[9] Rogosnitzky M., Danks R. (2011). Therapeutic potential of the biscoclaurine alkaloid, cepharanthine. Pharmacol. Rep..

[10] Nakase M., Nomura J., Inui M., Murata T., Kawarada Y., Tagawa T., Ohsugi H. (1997). Evaluation of clinical efficacy of Cepharanthin^®^ (gargle-internal use) treatment for oral mucosal lesions. J. Jpn. Oral Muco. Membr..

[11] Saki H., Ichihara H., Kato Y., Ando M., Abe K., Win K., Inoue T., Fujitsuka H., Hyodo I., Sugiyama T. (1994). Evaluation of clinical efficiency of Cepharanthin^®^ for the treatment of oral mucosal lesions and glossodynia. J. Jpn. Stomatol. Soc..

[12] Saito Y., Ikeda M., Tanaka H., Iijima J., Sakata K. (2001). A literatue study of oral therapeutics and pharmacology Report 1; Evidence of off-label use of cepharanthin. Oral. Ther. Pharmacol..

[13] Sogawa N., Sogawa C., Nakano M., Fukuoka R., Furuta H. (1998). Effects of propargylglycine on endotoxin-induced acute lethal toxicity and defensive effect of cepharanthin on this toxicity. J. Okayama Dent. Soc..

[14] Sogawa N., Sogawa C., Furuta H. (2000). A study of active ingredients in Cepharanthin^®^ on enhancement of lipopolysaccharide-induced histidine decarboxylase activities in mice spleens. Med. Biol..

[15] Yokota T., Yokota K., Matsuura T., Shiwa M. (1993). Suppressive effects of Cepharanthin^®^ on the production of superoxide anion by neutrophils during hemodialysis. J. Jpn. Soc. Dial. Ther..

[16] Sawamura D., Sato S., Suzuki M., Nomura K., Hanada K., Hashimoto I. (1988). Effect of cepharanthin on superoxide anion (O_2_^−^) production by macrophages. J. Dermatol..

[17] Akamatsu H., Komura J., Asada Y., Niwa Y. (1991). Effects of cepharanthin on neutrophil chemotaxis, phagocytosis, and reactive oxygen species generation. J. Dermatol..

[18] Kawada N., Mizoguchi Y., Kondo H., Seki S., Kobayashi K., Yamamoto S., Morisawa S. (1988). Effect of cepharanthine on metabolism of arachidonic acid from rat peritoneal exudate cells. Jpn. J. Inflamm..

[19] Kondo Y., Takano F., Hojo H. (1993). Inhibitory effect of bisbenzylisoquinoline alkaloids on nitric oxide production in activated macrophages. Biochem. Pharmacol..

[20] Matsuno T., Okazoe Y., kobayashi S., Obuchi H., Sato E., Edashige K., Utsumi K. (1989). Measurement of active oxygen of neutrophils by means of luminol chemiluminescence and their inhibition by biscoclaurine alkaloids. Igaku Yakugaku.

[21] Kondo Y., Takano F., Hojo H. (1993). Suppression of lipopolysaccharide-induced fulminant hepatitis and tumor necrosis factor production by bisbenzylisoquinoline alkaloids in bacillus Calmette-Guerin-treated mice. Biochem. Pharmacol..

[22] Sugiyama K., Sasaki J., Utsumi K., Miyahara M. (1976). Inhibition by cepharanthine of histamine release from rat peritoneal mast cells. Allergy.

[23] Sogawa N., Aoki-Sogawa C., Iwata-Abuku E., Inoue T., Oda N., Kishi K., Furuta H. (2001). Opposing pharmacological actions of cepharanthin on lipopolysaccharide-induced histidine decarboxylase activity in mice spleens. Life Sci..

[24] Noguchi K., Ishikawa I. (2007). The roles of cyclooxygenase-2 and prostaglandin E_2_ in periodontal disease. Periodontology 2000.

[25] Salvi G., Lang N. (2005). Host response modulation in the management of periodontal diseases. J. Clin. Periodontol..

[26] Ara T., Fujinami Y., Imamura Y., Wang P. (2008). Lipopolysaccharide-treated human gingival fibroblasts continuously produce PGE_2_. J. Hard Tissue Biol..

[27] Ara T., Kurata K., Hirai K., Uchihashi T., Uematsu T., Imamura Y., Furusawa K., Kurihara S., Wang P. (2009). Human gingival fibroblasts are critical in sustaining inflammation in periodontal disease. J. Periodontal. Res..

[28] Burke J., Dennis E. (2009). phospholipase A_2_ biochemistry. Cardiovasc Drugs Ther..

[29] Ara T., Sogawa N. (2016). Studies on shokyo, kanzo, and keihi in kakkonto medicine on prostaglandin E_2_ production in lipopolysaccharide-treated human gingival fibroblasts. Int. Sch. Res. Notices.

[30] Nemenoff R., Winitz S., Qian N., Van Putten V., Johnson G., Heasley L. (1993). Phosphorylation and activation of a high molecular weight form of phospholipase A_2_ by p42 microtubule-associated protein 2 kinase and protein kinase C. J. Biol. Chem..

[31] Lin L., Wartmann M., Lin A., Knopf J., Seth A., Davis R. (1993). cPLA_2_ is phosphorylated and activated by MAP kinase. Cell.

[32] Gijón M., Spencer D., Kaiser A., Leslie C. (1999). Role of phosphorylation sites and the C2 domain in regulation of cytosolic phospholipase A_2_. J. Cell. Biol..

[33] Gupta C., Katsumata M., Goldman A., Herold R., Piddington R. (1984). Glucocorticoid-induced phospholipase A_2_-inhibitory proteins mediate glucocorticoid teratogenicity in vitro. Proc. Natl. Acad. Sci. USA.

[34] Wallner B., Mattaliano R., Hession C., Cate R., Tizard R., Sinclair L., Foeller C., Chow E., Browing J., Ramachandran K. (1986). Cloning and expression of human lipocortin, a phospholipase A_2_ inhibitor with potential anti-inflammatory activity. Nature.

[35] Ara T., Fujinami Y., Urano H., Hirai K., Hattori T., Miyazawa H. (2012). Protein kinase A enhances lipopolysaccharide- induced IL-6, IL-8, and PGE_2_ production by human gingival fibroblasts. J. Negat. Results Biomed..

[36] Kitamura H., Urano H., Ara T. (2014). Preventive effects of a kampo medicine, kakkonto, on inflammatory responses via the suppression of extracellular signal-regulated kinase phosphorylation in lipopolysaccharide- treated human gingival fibroblasts. ISRN Pharmacol..

[37] Ara T., Maeda Y., Fujinami Y., Imamura Y., Hattori T., Wang P. (2008). Preventive effects of a Kampo medicine, Shosaikoto, on inflammatory responses in LPS-treated human gingival fibroblasts. Biol. Pharm. Bull..

[38] Nakazono Y., Ara T., Fujinami Y., Hattori T., Wang P. (2010). Preventive effects of a kampo medicine, hangeshashinto on inflammatory responses in lipopolysaccharide-treated human gingival fibroblasts. J. Hard Tissue Biol..

[39] Ara T., Sogawa N. (2017). Effects of shinbuto and ninjinto on prostaglandin E_2_ production in lipopolysaccharide- treated human gingival fibroblasts. PeerJ.

[40] Ara T., Honjo K., Fujinami Y., Hattori T., Imamura Y., Wang P. (2010). Preventive effects of a kampo medicine, orento on inflammatory responses in lipopolysaccharide treated human gingival fibroblasts. Biol. Pharm. Bull..

[41] Kato T., Segami N., Sakagami H. (2016). Anti-inflammatory activity of hangeshashinto in IL-1β-stimulated gingival and periodontal ligament fibroblasts. In Vivo.

[42] Miyamoto K., Lange M., McKinley G., Stavropoulos C., Moriya S., Matsumoto H., Inada Y. (1996). Effects of sho-saiko-to on production of prostaglandin E_2_ (PGE_2_), leukotriene B_4_ (LTB_4_) and superoxide from peripheral monocytes and polymorphonuclear cells isolated from HIV infected individuals. Am. J. Chin. Med..

[43] Horie N., Hashimoto K., Kato T., Shimoyama T., Kaneko T., Kusama K., Sakagami H. (2008). COX-2 as possible target for the inhibition of PGE_2_ production by Rikko-san in activated macrophage. In Vivo.

[44] Kaneko T., Chiba H., Horie N., Kato T., Hashimoto K., Kusama K., Sakagami H. (2008). Effect of Sairei-to and its ingredients on prostaglandin E_2_ production by mouse macrophage-like cells. In Vivo.

[45] Inoue M., Shen Y., Ogihara Y. (1996). Shosaikoto (kampo medicine) protects macrophage function from suppression by hypercholesterolemia. Biol. Pharm. Bull..

[46] Kase Y., Saitoh K., Ishige A., Komatsu Y. (1998). Mechanisms by which Hange-shashin-to reduces prostaglandin E2 levels. Biol. Pharm. Bull..

[47] Kase Y., Hayakawa T., Ishige A., Aburada M., Komatsu Y. (1997). The effects of *Hange-shashin-to* on the content of prostaglandin E_2_ and water absorption in the large intestine of rats. Biol. Pharm. Bull..

[48] Kase Y., Saitoh K., Yuzurihara M., Ishige A., Komatsu Y. (1998). Effects of *Hange-shashin-to* on cholera toxin-induced fluid secretion in the small intestine of rats. Biol. Pharm. Bull..

[49] Tjendraputra E., Tran V., Liu-Brennan D., Roufogalis B., Duke C. (2001). Effect of ginger constituents and synthetic analogues on cyclooxygenase-2 enzyme in intact cells. Bioorg. Chem..

[50] van Breemen R., Tao Y., Li W. (2011). Cyclooxygenase-2 inhibitors in ginger (*Zingiber officinale*). Fitoterapia.

[51] Lantz R., Chen G., Sarihan M., Solyom A., Jolad S., Timmermann B. (2007). The effect of extracts from ginger rhizome on inflammatory mediator production. Phytomedicine.

[52] Pan M., Hsieh M., Hsu P., Ho S., Lai C., Wu H., Sang S., Ho C. (2008). 6-Shogaol suppressed lipopolysaccharide- induced up-expression of iNOS and COX-2 in murine macrophages. Mol. Nutr. Food Res..

[53] Ha S., Moon E., Ju M., Kim D., Ryu J., Oh M., Kim S. (2012). 6-Shogaol, a ginger product, modulates neuroinflammation: A new approach to neuroprotection. Neuropharmacology.

[54] Kim S., Kundu J., Shin Y., Park J., Cho M., Kim T., Surh Y. (2005). [6]-Gingerol inhibits COX-2 expression by blocking the activation of p38 MAP kinase and NF-κB in phorbol ester-stimulated mouse skin. Oncogene.

[55] Saha P., Katarkar A., Das B., Bhattacharyya A., Chaudhuri K. (2016). 6-Gingerol inhibits *Vibrio cholerae*-induced proinflammatory cytokines in intestinal epithelial cells via modulation of NF-κB. Pharm. Biol..

[56] Ling H., Yang H., Tan S., Chui W., Chew E. (2010). 6-Shogaol, an active constituent of ginger, inhibits breast cancer cell invasion by reducing matrix metalloproteinase-9 expression via blockade of nuclear factor-κB activation. Br. J. Pharmacol..

[57] Nievergelt A., Marazzi J., Schoop R., Altmann K., Gertsch J. (2011). Ginger phenylpropanoids inhibit IL-1β and prostanoid secretion and disrupt arachidonate-phospholipid remodeling by targeting phospholipases A_2_. J. Immunol..

[58] Yu J., Ha J., Kim K., Jung Y., Jung J., Oh S. (2015). Anti-inflammatory activities of licorice extract and its active compounds, glycyrrhizic acid, liquiritin and liquiritigenin, in BV2 cells and mice liver. Molecules.

[59] Niwa K., Lian Z., Onogi K., Yun W., Tang L., Mori H., Tamaya T. (2007). Preventive effects of glycyrrhizin on estrogen-related endometrial carcinogenesis in mice. Oncol. Rep..

[60] Song J., Lee J., Shim B., Lee C., Choi S., Kang C., Sohn N., Shin J. (2013). Glycyrrhizin alleviates neuroinflammation and memory deficit induced by systemic lipopolysaccharide treatment in mice. Molecules.

[61] Takei H., Baba Y., Hisatsune A., Katsuki H., Miyata T., Yokomizo K., Isohama Y. (2008). Glycyrrhizin inhibits interleukin-8 production and nuclear factor-κB activity in lung epithelial cells, but not through glucocorticoid receptors. J. Pharmacol. Sci..

[62] Honda H., Nagai Y., Matsunaga T., Saitoh S., Akashi-Takamura S., Hayashi H., Fujii I., Miyake K., Muraguchi A., Takatsu K. (2012). Glycyrrhizin and isoliquiritigenin suppress the LPS sensor toll-like receptor 4/MD-2 complex signaling in a different manner. J. Leukoc. Biol..

[63] Takahashi T., Takasuka N., Iigo M., Baba M., Nishino H., Tsuda H., Okuyama T. (2004). Isoliquiritigenin, a flavonoid from licorice, reduces prostaglandin E_2_ and nitric oxide, causes apoptosis, and suppresses aberrant crypt foci development. Cancer Sci..

[64] Kim J., Park S., Yun K., Cho Y., Park H., Lee K. (2008). Isoliquiritigenin isolated from the roots of *Glycyrrhiza uralensis* inhibitsLPS-induced iNOS and COX-2 expression via the attenuation of NF-κB in RAW 264.7 macrophages. Eur. J. Pharmacol..

[65] Liao J., Deng J., Chiu C., Hou W., Huang S., Shie P., Huang G. (2012). Anti-inflammatory activities of *Cinnamomum cassia* constituents in vitro and in vivo. Evid. Based Complement. Alternat. Med..

[66] Yu T., Lee S., Yang W., Jang H., Lee Y., Kim T., Kim S., Lee J., Cho J. (2012). The ability of an ethanol extract of *Cinnamomum cassia* to inhibit Src and spleen tyrosine kinase activity contributes to its anti-inflammatory action. J. Ethnopharmacol..

[67] Kim B., Lee Y., Lee J., Lee J., Cho J. (2010). Regulatory effect of cinnamaldehyde on monocyte/macrophage- mediated inflammatory responses. Mediators Inflamm..

[68] Guo J., Huo H., Zhao B., Liu H., Li L., Ma Y., Guo S., Jiang T. (2006). Cinnamaldehyde reduces IL-1β-induced cyclooxygenase-2 activity in rat cerebral microvascular endothelial cells. Eur. J. Pharmacol..

[69] Youn H., Lee J., Choi Y., Saitoh S., Miyake K., Hwang D., Lee J. (2008). Cinnamaldehyde suppresses toll-like receptor 4 activation mediated through the inhibition of receptor oligomerization. Biochem. Pharmacol..

[70] Altavilla D., Squadrito F., Bitto A., Polito F., Burnett B., Di Stefano V., Minutoli L. (2009). Flavocoxid, a dual inhibitor of cyclooxygenase and 5-lipoxygenase, blunts pro-inflammatory phenotype activation in endotoxin-stimulated macrophages. Br. J. Pharmacol..

[71] Woo K., Lim J., Suh S., Kwon Y., Shin S., Kim S., Choi Y., Park J., Kwon T. (2006). Differential inhibitory effects of baicalein and baicalin on LPS-induced cyclooxygenase-2 expression through inhibition of C/EBPβ DNA-binding activity. Immunobiology.

[72] Seo M., Lee S., Jeon Y., Im J. (2011). Inhibition of p65 nuclear translocation by baicalein. Toxicol. Res..

[73] Chen Y., Shen S., Chen L., Lee T., Yang L. (2001). Wogonin, baicalin, and baicalein inhibition of inducible nitric oxide synthase and cyclooxygenase-2 gene expressions induced by nitric oxide synthase inhibitors and lipopolysaccharide. Biochem. Pharmacol..

[74] Pan M., Lai C., Wang Y., Ho C. (2006). Acacetin suppressed LPS-induced up-expression of iNOS and COX-2 in murine macrophages and TPA-induced tumor promotion in mice. Biochem. Pharmacol..

[75] Pandey M., Sung B., Kunnumakkara A., Sethi G., Chaturvedi M., Aggarwal B. (2008). Berberine modifies cysteine 179 of IκB*α* kinase, suppresses nuclear factor-κB-regulated antiapoptotic gene products, and potentiates apoptosis. Cancer Res..

[76] Jeong H., Hsu K., Lee J., Ham M., Huh J., Shin H., Kim W., Kim J. (2009). Berberine suppresses proinflammatory responses through AMPK activation in macrophages. Am. J. Physiol. Endocrinol. Metab..

[77] Lu D., Tang C., Chen Y., Wei I. (2010). Berberine suppresses neuroinflammatory responses through AMP-activated protein kinase activation in BV-2 microglia. J. Cell. Biochem..

[78] Kim H., Kim M., Kim E., Yang Y., Lee M., Lim J. (2012). Berberine-induced AMPK activation inhibits the metastatic potential of melanoma cells via reduction of ERK activity and COX-2 protein expression. Biochem. Pharmacol..

[79] Liang K., Ting C., Yin S., Chen Y., Lin S., Liao J., Hsu S. (2006). Berberine suppresses MEK/ERK-dependent Egr-1 signaling pathway and inhibits vascular smooth muscle cell regrowth after in vitro mechanical injury. Biochem. Pharmacol..

[80] Afzal M., Al-Hadidi D., Menon M., Pesek J., Dhami M. (2001). Ginger: An ethnomedical, chemical and pharmacological review. Drug Metabol. Drug Interact..

[81] Lakhan S., Ford C., Tepper D. (2015). *Zingiberaceae* extracts for pain: A systematic review and meta-analysis. Nutr. J..

[82] Thomson M., Al-Qattan K., Al-Sawan S., Alnaqeeb M., Khan I., Ali M. (2002). The use of ginger (*Zingiber officinale* Rosc.) as a potential anti-inflammatory and antithrombotic agent. Prostaglandins Leukot Essent Fatty Acids.

[83] Aimbire F., Penna S., Rodrigues M., Rodrigues K., Lopes-Martins R., Sertié J. (2007). Effect of hydroalcoholic extract of *Zingiber officinalis* rhizomes on LPS-induced rat airway hyperreactivity and lung inflammation. Prostaglandins Leukot Essent Fatty Acids.

[84] El-Abhar H., Hammad L., Gawad H. (2008). Modulating effect of ginger extract on rats with ulcerative colitis. J. Ethnopharmacol..

[85] Podlogar J., Verspohl E. (2012). Antiinflammatory effects of ginger and some of its components in human bronchial epithelial (BEAS-2B) cells. Phytother. Res..

[86] Shim S., Kim S., Choi D., Kwon Y., Kwon J. (2011). Anti-inflammatory effects of [6]-shogaol: Potential roles of HDAC inhibition and HSP70 induction. Food Chem. Toxicol..

[87] Shibata S. (2000). A drug over the millennia: Pharmacognosy, chemistry, and pharmacology of licorice. Yakugaku Zasshi.

[88] Farese R., Biglieri E., Shackleton C., Irony I., Gomez-Fontes R. (1991). Licorice-induced hypermineralocorticoidism. N. Engl. J. Med..

[89] Mumoli N., Cei M. (2008). Licorice-induced hypokalemia. Int. J. Cardiol..

[90] Van Uum S. (2005). Liquorice and hypertension. Neth. J. Med..

[91] Palermo M., Quinkler M., Stewart P. (2004). Apparent mineralocorticoid excess syndrome: An overview. Arq. Bras. Endocrinol. Metabol..

[92] van Uum S., Lenders J., Hermus A. (2004). Cortisol, 11β-hydroxysteroid dehydrogenases, and hypertension. Semin. Vasc. Med..

[93] Walker B., Edwards C. (1994). Licorice-induced hypertension and syndromes of apparent mineralocorticoid excess. Endocrinol. Metab. Clin. N. Am..

[94] Kaneko T., Chiba H., Horie N., Kato T., Kobayashi M., Hashimoto K., Kusama K., Sakagami H. (2009). Effect of Scutellariae radix ingredients on prostaglandin E_2_ production and COX-2 expression by LPS-activated macrophage. In Vivo.

[95] Kuo C., Chi C., Liu T. (2004). The anti-inflammatory potential of berberine in vitro and in vivo. Cancer Lett..

[96] Kuo C., Chi C., Liu T. (2005). Modulation of apoptosis by berberine through inhibition of cyclooxygenase-2 and Mcl-1 expression in oral cancer cells. In Vivo.

[97] Liang Y., Huang B., Song E., Bai B., Wang Y. (2014). Constitutive activation of AMPK *α*1 in vascular endothelium promotes high-fat diet-induced fatty liver injury: Role of COX-2 induction. Br. J. Pharmacol..

[98] Leech M., Bartold P. (2015). The association between rheumatoid arthritis and periodontitis. Best Pract. Res. Clin. Rheumatol..

[99] De Pablo P., Dietrich T., McAlindon T. (2008). Association of periodontal disease and tooth loss with rheumatoid arthritis in the US population. J. Rheumatol..

[100] Zhao X., Liu Z., Shu D., Xiong Y., He M., Xu S., Si S., Guo B. (2018). Association of periodontitis with rheumatoid arthritis and the effect of non-surgical periodontal treatment on disease activity in patients with rheumatoid arthritis. Med. Sci. Monit..

[101] Araújo V., Melo I., Lima V. (2015). Relationship between periodontitis and rheumatoid arthritis: Review of the literature. Mediators Inflamm..

[102] Kaur S., Bright R., Proudman S., Bartold P. (2014). Does periodontal treatment influence clinical and biochemical measures for rheumatoid arthritis? A systematic review and meta-analysis. Semin. Arthritis Rheum..

[103] Javed F., Ahmed H., Mikami T., Almas K., Romanos G., Al-Hezaimi K. (2014). Cytokine profile in the gingival crevicular fluid of rheumatoid arthritis patients with chronic periodontitis. J. Investig. Clin. Dent..

[104] Erciyas K., Sezer U., Ustün K., Pehlivan Y., Kisacik B., Senyurt S., Tarakçioğlu M., Onat A. (2013). Effects of periodontal therapy on disease activity and systemic inflammation in rheumatoid arthritis patients. Oral Dis..

[105] Gümüş P., Buduneli E., Bıyıkoğlu B., Aksu K., Saraç F., Nile C., Lappin D., Buduneli N. (2013). Gingival crevicular fluid, serum levels of receptor activator of nuclear factor-κB ligand, osteoprotegerin, and interleukin-17 in patients with rheumatoid arthritis and osteoporosis and with periodontal disease. J. Periodontol..

[106] Silosi I., Cojocaru M., Foia L., Boldeanu M., Petrescu F., Surlin P., Biciusca V. (2015). Significance of circulating and crevicular matrix metalloproteinase-9 in rheumatoid arthritis-chronic periodontitis association. J. Immunol. Res..

[107] Li J., Huang Z., Wang R., Ma X., Zhang Z., Liu Z., Chen Y., Su Y. (2013). Fruit and vegetable intake and bone mass in Chinese adolescents, young and postmenopausal women. Public Health Nutr..

[108] Hardcastle A., Aucott L., Fraser W., Reid D., Macdonald H. (2011). Dietary patterns, bone resorption and bone mineral density in early post-menopausal Scottish women. Eur. J. Clin. Nutr..

[109] Uchiyama S., Yamaguchi M. (2004). Inhibitory effect of beta-cryptoxanthin on osteoclast-like cell formation in mouse marrow cultures. Biochem. Pharmacol..

[110] Matsumoto C., Ashida N., Yokoyama S., Tominari T., Hirata M., Ogawa K., Sugiura M., Yano M., Inada M., Miyaura C. (2013). The protective effects of β-cryptoxanthin on inflammatory bone resorption in a mouse experimental model of periodontitis. Mol. Med. Rep..

[111] Zeng W., Jin L., Zhang F., Zhang C., Liang W. (2018). Naringenin as a potential immunomodulator in therapeutics. Pharmacol. Res..

[112] Li Y., Chen D., Chu C., Li S., Chen Y., Wu C., Lin C. (2015). Naringenin inhibits dendritic cell maturation and has therapeutic effects in a murine model of collagen-induced arthritis. J. Nutr. Biochem..

[113] La V., Tanabe S., Grenier D. (2009). Naringenin inhibits human osteoclastogenesis and osteoclastic bone resorption. J. Periodontal. Res..

[114] Kimira Y., Taniuchi Y., Nakatani S., Sekiguchi Y., Kim H., Shimizu J., Ebata M., Wada M., Matsumoto A., Mano H. (2015). Citrus limonoid nomilin inhibits osteoclastogenesis in vitro by suppression of NFATc1 and MAPK signaling pathways. Phytomedicine.

[115] Gu L., Deng W., Liu Y., Jiang C., Sun L., Sun X., Xu Q., Zhou H. (2014). Ellagic acid protects Lipopolysaccharide/ D-galactosamine-induced acute hepatic injury in mice. Int. Immunopharmacol..

[116] Beserra A., Calegari P., Souza Mdo C., Dos Santos R., Lima J., Silva R., Balogun S., Martins D. (2011). Gastroprotective and ulcer-healing mechanisms of ellagic acid in experimental rats. J. Agric. Food Chem..

[117] Marín M., María Giner R., Ríos J., Recio M. (2013). Intestinal anti-inflammatory activity of ellagic acid in the acute and chronic dextrane sulfate sodium models of mice colitis. J. Ethnopharmacol..

[118] Mo J., Panichayupakaranant P., Kaewnopparat N., Songkro S., Reanmongkol W. (2014). Topical anti-inflammatory potential of standardized pomegranate rind extract and ellagic acid in contact dermatitis. Phytother. Res..

[119] Allam G., Mahdi E., Alzahrani A., Abuelsaad A. (2016). Ellagic acid alleviates adjuvant induced arthritis by modulation of pro- and anti-inflammatory cytokines. Cent. Eur. J. Immunol..

[120] Granica S., Kłębowska A., Kosiński M., Piwowarski J., Dudek M., Kaźmierski S., Kiss A. (2016). Effects of *Geum urbanum* L. root extracts and its constituents on polymorphonuclear leucocytes functions. Significance in periodontal diseases. J. Ethnopharmacol..

[121] Ahmed S., Pakozdi A., Koch A. (2006). Regulation of interleukin-1β-induced chemokine production and matrix metalloproteinase 2 activation by epigallocatechin-3-gallate in rheumatoid arthritis synovial fibroblasts. Arthritis Rheum..

[122] Yun H., Yoo W., Han M., Lee Y., Kim J., Lee S. (2008). Epigallocatechin-3-gallate suppresses TNF-*α*-induced production of MMP-1 and -3 in rheumatoid arthritis synovial fibroblasts. Rheumatol. Int..

[123] Ahmed S., Marotte H., Kwan K., Ruth J., Campbell P., Rabquer B., Pakozdi A., Koch A. (2008). Epigallocatechin-3-gallate inhibits IL-6 synthesis and suppresses transsignaling by enhancing soluble gp130 production. Proc. Natl. Acad. Sci. USA.

[124] Gadagi J., Chava V., Reddy V. (2013). Green tea extract as a local drug therapy on periodontitis patients with diabetes mellitus: A randomized case-control study. J. Indian Soc. Periodontol..

[125] Sekiguchi Y., Mano H., Nakatani S., Shimizu J., Wada M. (2010). Effects of the Sri Lankan medicinal plant, *Salacia reticulata*, in rheumatoid arthritis. Genes Nutr..

[126] Sekiguchi Y., Mano H., Nakatani S., Shimizu J., Kataoka A., Ogura K., Kimira Y., Ebata M., Wada M. (2017). Mangiferin positively regulates osteoblast differentiation and suppresses osteoclast differentiation. Mol. Med. Rep..

